# PhoSARte: identification of SARS-CoV-2 phosphorylation sites using contrastive learning and protein language models

**DOI:** 10.1093/bib/bbag504

**Published:** 2026-09-15

**Authors:** Nhat Truong Pham, Duong Thanh Tran, Qiaosen Su, Yeona Jung, Nattanong Bupi, Dahyun Kang, Sukchan Lee, Balachandran Manavalan

**Affiliations:** Department of Integrative Biotechnology, College of Biotechnology and Bioengineering, Sungkyunkwan University, Suwon 16419, Gyeonggi-do, Republic of Korea; Department of Integrative Biotechnology, College of Biotechnology and Bioengineering, Sungkyunkwan University, Suwon 16419, Gyeonggi-do, Republic of Korea; Department of Integrative Biotechnology, College of Biotechnology and Bioengineering, Sungkyunkwan University, Suwon 16419, Gyeonggi-do, Republic of Korea; Department of Integrative Biotechnology, College of Biotechnology and Bioengineering, Sungkyunkwan University, Suwon 16419, Gyeonggi-do, Republic of Korea; Department of Integrative Biotechnology, College of Biotechnology and Bioengineering, Sungkyunkwan University, Suwon 16419, Gyeonggi-do, Republic of Korea; Department of Integrative Biotechnology, College of Biotechnology and Bioengineering, Sungkyunkwan University, Suwon 16419, Gyeonggi-do, Republic of Korea; Department of Integrative Biotechnology, College of Biotechnology and Bioengineering, Sungkyunkwan University, Suwon 16419, Gyeonggi-do, Republic of Korea; Department of Integrative Biotechnology, College of Biotechnology and Bioengineering, Sungkyunkwan University, Suwon 16419, Gyeonggi-do, Republic of Korea

**Keywords:** contrastive learning, pretrained protein language models, protein post-translational modification, phosphorylation sites, SARS-CoV-2-infected cells, Siamese network

## Abstract

Phosphorylation, a critical post-translational modification, is extensively altered during viral infections, including SARS-CoV-2, where it plays a central role in modulating host–pathogen interactions. Accurately identifying these specific phosphorylation sites is crucial for understanding viral pathogenesis, prioritizing antiviral targets, guiding therapeutic strategies, and strengthening preparedness for future viral outbreaks. Although several computational tools have been proposed to complement experimental phosphoproteomics, existing methods often show limited robustness, cross-viral generalizability, and interpretability. To address these challenges, we developed PhoSARte, an interpretable computational framework that integrates Siamese network-based contrastive learning (SCL) with pretrained protein language models (PLMs) to accurately identify phosphorylation sites in SARS-CoV-2-infected cells. PhoSARte employs a unique dual-stream architecture: PLMs capture contextual protein sequence representations, while the SCL module, comprising a transformer-based encoder and an attention-based bidirectional gated recurrent unit, learns discriminative and similarity-preserving representations of protein sequence pairs using a contrastive loss function. The integration of these complementary representations substantially improves the robustness and generalizability of the framework. PhoSARte was rigorously benchmarked on phosphoproteomics datasets derived from infected A549 (*Homo sapiens*) and Vero E6 (*Chlorocebus sabaeus*) cells, as well as their combined dataset. Through rigorous cross-cell-type validation and testing, PhoSARte demonstrated superior performance, significantly outperforming current state-of-the-art methods. Importantly, an external cross-viral case study on entirely unseen adenovirus type 2-infected human IMR-90 cells confirmed the broad transferability of PhoSARte, demonstrating its capacity to generate actionable biological hypotheses under novel viral stress conditions. Furthermore, advancing beyond traditional black-box predictors, PhoSARte integrates an *in silico* mutagenesis analysis that decodes complex deep learning embeddings to successfully extract biologically relevant motif signatures. PhoSARte is freely accessible at https://balalab-skku.org/PhoSARte/, providing an accessible, interpretable, and adaptable framework for virus-associated phosphorylation site prediction, antiviral target prioritization, and host-directed therapeutic discovery.

## Introduction

Protein phosphorylation is a fundamental and widespread post-translational modification [1] that dynamically regulates multiple cellular processes, including signaling, metabolism, and immune responses. This modification involves the transfer of a phosphate group from adenosine triphosphate to specific amino acid residues in proteins, catalyzed by protein kinases. Phosphorylation predominantly occurs on serine (S) and threonine (T) residues, which together account for more than 98% of known phosphorylation sites [2]. By modulating protein activity, stability, conformation, and protein–protein interactions, phosphorylation orchestrates essential physiological processes such as growth signal response, cell cycle progression, cellular stress response, neuronal function, and immune response [1].

The COVID-19 pandemic, caused by severe acute respiratory syndrome coronavirus 2 (SARS-CoV-2), revealed how viral pathogens exploit phosphorylation to manipulate host cellular pathways. SARS-CoV-2 hijacks host kinase signaling networks to enhance replication and suppress antiviral responses [3]. This virus-driven reprogramming is extensive, with approximately 70 phosphorylation sites identified in viral proteins and more than 15 000 phosphorylation events mapped in host proteins during infection [4]. Of the 29 viral proteins encoded by the SARS-CoV-2 genome, four major structural proteins, namely spike, envelope, membrane, and nucleocapsid, are key to infection [5]. Among these, the nucleocapsid protein is crucial for viral RNA replication, genome packaging, and interactions with the host [6]. Notably, Yaron *et al.* showed that multiple phosphorylation sites clustered within a conserved region of the viral nucleocapsid protein, directly affecting viral replication in infected A549 (*Homo sapiens*) and Vero E6 (*Chlorocebus sabaeus*) cells [7]. These findings highlight that identifying phosphorylation sites in both host and viral proteins is vital for understanding the molecular mechanisms of SARS-CoV-2 infection and may uncover new therapeutic targets. Recent *in silico* studies on emerging viral pathogens have further demonstrated that integrative computational pipelines combining molecular docking, molecular dynamics simulations, and absorption, distribution, metabolism, excretion, and toxicity profiling can systematically characterize viral protein structure–function relationships, as shown for monkeypox and Marburg virus [8] and human T-cell leukemia virus 1 (HTLV-1)-associated lymphoma [9]. Similar multi-step computational workflows, encompassing docking, dynamics, pharmacokinetics, and quantum calculations, have been successfully applied to virus-associated targets, including human papillomavirus oncoproteins [10] and antiparasitic discovery against *Babesia microti* [11].

Over the past decade, mass spectrometry (MS)-based proteomics methods have been used to identify phosphorylated proteins and peptides with high throughput, resolution, and accuracy by detecting changes in protein levels and phosphorylation, especially when these changes are caused by SARS-CoV-2 infection [12]. For example, Bojkova *et al.* used proteomics analysis to identify host cell pathways affected by SARS-CoV-2 and showed that blocking these pathways can prevent viral replication in human cells [12]. Stukalov *et al.* employed advanced proteomics techniques to map the interactomes of SARS-CoV-2 and examine its effects on the transcriptome, proteome, ubiquitinome, and phosphoproteome of a lung-derived human cell line (A549) [13]. Bouhaddou *et al.* carried out a quantitative MS-based phosphoproteomics study of SARS-CoV-2 infection in Vero E6 (*C. sabaeus*) cells, revealing significant changes in protein phosphorylation patterns within host cells [14]. Similarly, Hekman *et al.* conducted a comparable quantitative MS-based phosphoproteomics study to investigate the mechanisms of infection and pathology caused by SARS-CoV-2 in induced pluripotent stem cell-derived alveolar epithelial type 2 (iAT2) cells [15]. Klann *et al.* used MS-based phosphoproteomics to analyze signaling changes triggered by SARS-CoV-2 infection in human Caco-2 cells [16]. However, these experimental strategies are often time-consuming and expensive, especially when analyzing large datasets. Nonetheless, they offer detailed annotations of phosphorylation sites that are vital for developing computational, machine learning (ML), and deep learning (DL) methods.

Recently, several computational methods and ML- and DL-based approaches have been developed to identify phosphorylation sites in SARS-CoV-2-infected cells. DeepIPs was the first method proposed by Lv *et al.* [17], in which they utilized A549 (*H. sapiens*) cells and developed a DL-based framework using convolutional neural networks combined with long short-term memory (LSTM) to learn and extract high-level motif features and long-range dependencies for the final prediction. Subsequently, several methods have been developed utilizing this dataset, such as DE-MHAIPs [18], IPs-GRUAtt [19], MeL-STPhos [20], PSPred-ALE [21], LGB-IPs [22], Deepph [23], PhosBERT [24], PTransIPs [25], Res-GCN [26], and GBMPhos [27]. Although these methods have significantly advanced the field, they mostly rely on either handcrafted features or DL-based feature extraction, which may limit their ability to achieve optimal predictive performance, generalizability across different cell types, and robustness under unseen infection conditions. This limitation is particularly important because, although the acute phase of the COVID-19 pandemic has subsided, the evolutionary progression from SARS-CoV to SARS-CoV-2 highlights the persistent risk posed by coronaviruses and future zoonotic spillovers [28, 29]. Therefore, accurate identification of phosphorylation-driven host–pathogen interactions remains essential not only for current therapeutic development but also for establishing a biomedical baseline for preparedness against emerging viral threats [14, 30]. These challenges highlight the need for more expressive, robust, and transferable frameworks capable of capturing the complex phosphorylation-associated sequence patterns across both viral and host proteins. In this context, protein language models (PLMs) combined with contrastive learning offer a promising alternative for data-driven representation learning from protein sequences, thereby enabling the identification of phosphorylation-specific signatures in both SARS-CoV-2 and host proteins without manual feature engineering.

**Figure 1:**
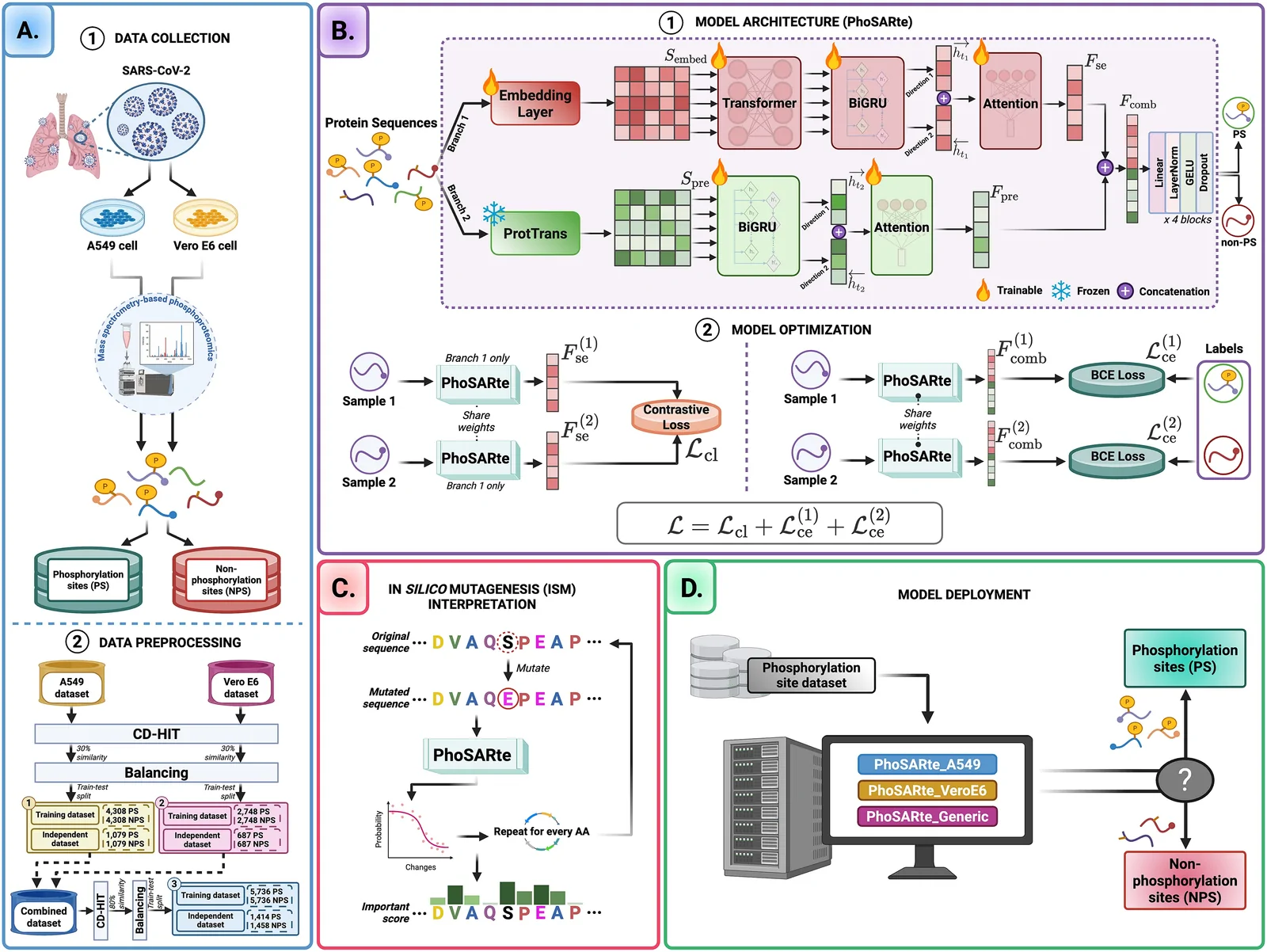
Overview of the PhoSARte framework. (A) Phosphorylation sites were gathered from SARS-CoV-2-infected cell lines, namely A549 and Vero E6, and a combined dataset was created by integrating these cell lines. All these datasets were processed with CD-HIT to reduce redundancy and balance the samples by selecting an equal number of phosphorylated and non-phosphorylated sites, and then split into training and testing datasets. (B) Next, the protein sequences were passed through SCL and pretrained PLM modules to learn and extract discriminative feature representations and PLM-based embeddings, respectively. These features were then fused through concatenation and fed into a classification head module for final prediction. (C) Model interpretation was performed using *in silico* mutagenesis (ISM) analysis. (D) During the testing phase, phosphorylation site datasets were fed into the trained models to predict either phosphorylated sites or non-phosphorylated sites. (*Created with Flaticon.com and BioRender.com.*)

In this study, we introduce PhoSARte, the first computational framework to combine Siamese network-based contrastive learning (SCL) with pretrained PLMs for accurate identification of S/T phosphorylation sites in SARS-CoV-2-infected cells (Fig. 1). The primary methodological novelty of PhoSARte lies in its dual-stream architecture. Unlike previous approaches that mainly rely on handcrafted descriptors or conventional feature extraction, PhoSARte explicitly integrates SCL and PLM modules to enhance the separation between positive and negative phosphorylation sequences under infected-cell conditions. Specifically, the SCL branch, consisting of a transformer-based encoder (TransEnc) and an attention-based bidirectional gated recurrent unit (AttBiGRU), learns discriminative and similarity-preserving representations of protein sequence pairs using a contrastive loss function. Meanwhile, the PLM module captures contextual embeddings of protein sequences, which are then fed into another AttBiGRU to obtain high-level feature representations. These complementary feature representations are then integrated by concatenation to improve the feature space, enabling more robust identification of S/T phosphorylation sites. PhoSARte consistently outperforms other models and existing methods in both five-fold cross-validation and testing, demonstrating strong transferability. To further assess its broader applicability beyond SARS-CoV-2, we conducted a cross-viral case study using an entirely unseen adenovirus type 2-infected cell dataset. This analysis demonstrates the potential of PhoSARte to prioritize infection-associated phosphorylation events under different viral stress conditions and to support hypothesis generation in host–pathogen interaction studies. Furthermore, to address the black-box limitation of many ML-based and DL-based predictors, PhoSARte incorporates *in silico* mutagenesis (ISM)-based interpretation to identify sequence positions and residue preferences that drive model predictions. PhoSARte is freely available at https://balalab-skku.org/PhoSARte/, providing accessible and interpretable resources for identifying phosphorylation sites and analyzing host–virus interactions.

## Materials and methods

### Dataset collection

To ensure a comprehensive and fair benchmarking, we utilized three well-established phosphorylation site datasets introduced by Lv *et al.* and our previous study [17, 20], encompassing A549 (*H. sapiens*), Vero E6 (*C. sabaeus*), and a combination of both cells (both species) (Fig. 1A). These datasets were specifically designed to reflect phosphorylation events in the context of SARS-CoV-2 infection and have been widely used to develop existing predictors.

(i) The first dataset, based on the A549 human cell line (A549), was originally constructed by Stukalov *et al.* using experimentally validated phosphorylation sites from SARS-CoV-2-infected A549 cells [13]. This dataset was subsequently used to develop the DeepIPs predictor [17]. Notably, the dataset comprises 14 119 experimentally verified phosphorylation sites on S, T, and tyrosine (Y). It was then processed into 33-residue segments centered on the phosphorylation site. To ensure data quality and reduce redundancy, they employed CD-HIT [31] with a 30% sequence identity threshold. The S/T dataset was then balanced by randomly selecting negative (non-phosphorylated) samples, resulting in 5387 positive and 5387 negative samples. This was then partitioned into a training set (4308 positive and 4308 negative samples) and a testing set (1079 positive and 1079 negative samples). The same study constructed a dataset for Y phosphorylation sites. However, due to the extremely low prevalence of these Y phosphorylation sites (1.80%) compared to S (86.40%) and T (11.80%), as previously reported [2], this dataset is too small to develop a prediction model, and thus, we excluded it from our analysis.

(ii) In our previous study on developing the MeL-STPhos [20], we constructed a Vero E6 dataset using experimentally validated phosphorylation sites from SARS-CoV-2-infected African green monkey Vero E6 cells, as reported by Bouhaddou *et al.* [14]. Notably, Bouhaddou *et al.* [14] filtered phosphorylated sites in *C. sabaeus* that align with orthologous *H. sapiens* S/T positions and provided these filtered phosphorylated sites in their supplementary information (“PhosphoDataFiltered” sheet). Following the same windowing strategy as Lv *et al.* [17], we extracted 33-residue sequence segments centered on S/T phosphorylation sites, excluding C- and N-terminal sites. To reduce sequence redundancy, we applied CD-HIT with a 30% sequence identity threshold, resulting in 3435 phosphorylation sites and 191 008 non-phosphorylation sites. An equal number of negative samples was randomly selected to balance the positive samples, resulting in a final split of 2748 positive and 2748 negative samples for training, and 687 positive and 687 negative samples for testing. As with the A549 dataset, we focused exclusively on S/T phosphorylation sites due to the low prevalence and poor representation of Y sites in the sourcedata.

(iii) The final dataset (Combined) was constructed in MeL-STPhos [20] by integrating the training and testing sets from the A549 and Vero E6 datasets, respectively. In this study, we used the same filtered dataset provided by Bouhaddou *et al.* [14] to construct the Vero E6 dataset, thereby ensuring positional correspondence between the A549 and Vero E6 datasets when building the Combined dataset. This strategy minimized ambiguity caused by cell line-specific phosphorylation differences and enabled integration based on comparable orthologous phosphorylation sites. To further reduce redundancy, CD-HIT was applied with an 80% sequence identity threshold, and all overlapping sequences between training and test sets were excluded to prevent information leakage. The resulting dataset consisted of 5736 positive and 5736 negative samples for training, and 1414 positive and 1458 negative samples for testing.

To ensure consistency with previous predictors and enable fair performance comparison, each protein sequence from the three datasets was truncated into 33-residue segments centered on the phosphorylation site, following the windowing strategy of Lv *et al.* [17]. This window size was selected based on the previous study by Huang *et al.* [27], who systematically compared multiple sequence lengths and demonstrated that a 33-residue window achieved the best predictive performance, thereby providing a strong rationale for its use in this study. Additionally, the training sets were used exclusively for hyperparameter optimization through five-fold cross-validation, whereas the testing sets were solely used for evaluating the effectiveness and robustness of the trained models. Details of the data preparation process, including the construction of positive and negative sequence pairs, are provided in Supplementary Note S1.

### Proposed architecture of PhoSARte

The PhoSARte framework integrates two complementary branches for effective feature representation learning (Fig. 1B): an SCL module (Branch 1) and a PLM module (Branch 2). The outputs of both branches are subsequently fused and fed into a classification head module for final prediction. The architecture and functionality of each module are as follows:

### Branch 1: Siamese network-based contrastive learning module

The SCL module is designed to learn the similarity-preserving and dissimilarity representations. The architecture employs two identical subnetworks with shared parameters to process paired input sequences and project them into a common feature space, where a contrastive loss function pulls similar pairs to cluster closely while pushing dissimilar pairs apart (Fig. 1B). Our SCL design mainly consists of three components: a TransEnc, an AttBiGRU, and contrastive learning.

#### Transformer-based encoder

The TransEnc is the first stage of the SCL module and is designed to capture contextual relationships and long-range dependencies within protein sequences [32]. We chose this method because of its proven ability in natural language processing to handle sequence-to-sequence tasks effectively. Similar to how a transformer tracks the relationship between words in a sentence, it learns contextual semantic information by analyzing the relationships between amino acids in a protein sequence.

A protein is a chain of amino acids, represented as a sequence $S=\left({a}_1,{a}_2,\dots, {a}_n\right)$, where ${a}_i$ is the *i*-th amino acid and *n* defines the sequence length. To prepare the sequence for the model, we constructed a vocabulary by mapping each amino acid to a specific index. For instance, A (alanine) becomes 1, R (arginine) becomes 2, N (asparagine) becomes 3, and so on. This strategy converts a sequence like “MCAD…A” into a numerical vector such as [13 5 1 4…1]. The vector is then fed into a learnable embedding layer to transform it into a more meaningful embedding matrix (${\mathrm{S}}_{\mathrm{embed}}$).

The embedding matrix ${\mathrm{S}}_{\mathrm{embed}}$ is then processed by a multi-headed self-attention (MSA) block. The mechanism allows the model to consider all other residues in the input sequence when encoding a specific residue. The output from the self-attention layer is then passed to a feed-forward network (FFN) block. Both layers incorporate a residual connection and layer normalization (LN) to stabilize training and improve performance. The output ${S}_{output}^i$ of the *i*-th encoder layer is defined as:


(1) \begin{equation*} {S}_{\mathrm{output}}^i=\mathrm{MSA}\left(\mathrm{LN}\left({S}_{\mathrm{output}}^{i-1}\right)\right)+{S}_{\mathrm{output}}^{i-1} \end{equation*}

(2) \begin{equation*} {S}_{output}^i=\mathrm{FFN}\left(\mathrm{LN}\left({S}_{output}^i\right)\right)+{S}_{output}^i \end{equation*}

When *i=1*, ${S}_{output}^{i-1}$ = ${S}_{embed}.$This process ultimately generates a rich, context-dependent feature representation of the sequence, capturing local and global information, which is crucial for the subsequent BiGRU framework.

#### Attention-based BiGRU

The GRU is a specialized type of recurrent neural network (RNN) that addresses the problem of vanishing or exploding gradients in traditional RNNs [33]. It is a simplified, more efficient variant of the LSTM network [34], resulting in faster computation with fewer parameters. Detailed information about the GRU’s architecture is included in Supplementary Note S2.

In natural language processing, not all words contribute equally to the meaning of a sentence [35]. Similarly, in protein sequences, not all amino acids contribute equally to the characterization of phosphorylation sites. Therefore, we incorporated an attention mechanism [36–39] on top of the BiGRU output to enhance model performance by assigning weights to the most informative amino acids at each position. This enables our model to learn biologically relevant sequence motifs associated with phosphorylation. The mathematical equations for this attention mechanism are provided in Supplementary Note S2.

#### Contrastive learning

To effectively learn a representational embedding space, we employed contrastive learning to learn the similarity between input feature vectors. This approach aims to create a space where similar protein sequences are clustered together, while dissimilar ones are pushed further apart. For each input pair, the SCL module generated corresponding representations ${\mathrm{F}}_{se}^{(1)}$ and ${\mathrm{F}}_{se}^{(2)}$. Subsequently, Euclidean distance was adopted to measure the similarity of the two inputs as follows:


(3) \begin{equation*} D\left({\mathrm{F}}_{se}^{(1)},{\mathrm{F}}_{se}^{(2)}\right)={\parallel {\mathrm{F}}_{se}^{(1)}-{\mathrm{F}}_{se}^{(2)}\parallel}_2 \end{equation*}

where the representation of the sequence *S* generated by the Siamese network was obtained as follows:


(4) \begin{equation*} {F}_{se}^{(i)}=\mathrm{AttBiGRU}\left(\mathrm{TransEnc}(S)\right) \end{equation*}

To train the model, we utilized a contrastive loss function [40], which allows embeddings of similar sequences to remain close and dissimilar ones to remain at least a margin *m* apart. The loss function is defined as:


(5) \begin{equation*} {\mathcal{L}}_{cl}=\frac{1}{2}\left(1-K\right)D{\left({\mathrm{F}}_{se}^{(1)},{\mathrm{F}}_{se}^{(2)}\right)}^2+\frac{1}{2}K\left\{\max {\left(0,m-D\left({\mathrm{F}}_{se}^{(1)},{\mathrm{F}}_{se}^{(2)}\right)\right)}^2\right\} \end{equation*}

Here, *K=0* if the input pair (${S}_1$ and ${S}_2$) belongs to the same class (i.e. both phosphorylated or non-phosphorylated), and *K=1* otherwise. *D* is the Euclidean distance between their representations ${\mathrm{F}}_{se}^{(1)}$ and ${\mathrm{F}}_{se}^{(2)}$, and *m* is a margin hyperparameter (*m>0*). If *D>m* for dissimilar pairs, no penalty was applied. Otherwise, the model was optimized to increase the distance between representations. This contrastive learning objective allows the model to learn semantically meaningful representations of input pairs, and the network parameters are updated accordingly via backpropagation. Supplementary Notes S1 and S2 and Table S1 available online at http://bib.oxfordjournals.org/ contain detailed information about the SCL module.

### Branch 2: protein language model module

To capture high-level semantic and biophysical information from protein sequences, we utilized the ProtTrans [41] pretrained language model family. These models, trained in an unsupervised fashion on massive protein corpora, are capable of extracting rich contextual embeddings. We experimented with different ProtTrans variants, including Prot-BERT-BFD, Prot-T5-XL-BFD, Prot-XLNet, Prot-Albert, Prot-T5-XL-Uniref50, Prot-T5-XXL-Uniref50, Prot-BERT, and Prot-T5-XXL-BFD, to extract semantic information. For each protein sequence *S*, the PLM module yielded a feature embedding ${S}_{pre}\in {\mathbb{R}}^{L\times E}$, where *L* is the sequence length and *E* is the embedding dimension ($E\in \left\{1024,4096\right\}$).

These embeddings were then fed into another AttBiGRU architecture to obtain a higher-level feature representation (${F}_{pre}$). It should be noted that this AttBiGRU differs from the one used in the SCL module in terms of both the number of recurrent layers and the expected feature dimension. Supplementary Table S2 contains detailed information about the PLM module.

### Classification head module

The features learned from the SCL module (${F}_{se}$) were concatenated with the features extracted from the PLM module (${F}_{pre}$) to form a combined feature vector (${F}_{comb}$). This integrated representation was then fed into a fully connected network (FCN) layer for the final prediction. The final prediction (${y}_{\mathrm{pred}}$) is determined by the following:


(6) \begin{equation*} {y}_{pred}=\mathrm{FCN}\left({F}_{comb}\right) \end{equation*}

Here, we adopted a binary cross-entropy function as the loss function as follows:


(7) \begin{equation*} {\mathcal{L}}_{\mathrm{ce}}=-\left(y\log \left({y}_{\mathrm{pred}}\right)-\left(1-y\right)\log \left(1-{y}_{\mathrm{pred}}\right)\right. \end{equation*}

where *y*∈{0,1} is the label and ${y}_{pred}$ is the predicted probability. The final loss function used to optimize the entire model is a combination of contrastive loss (${\mathcal{L}}_{cl}$) and binary cross-entropy (${\mathcal{L}}_{\mathrm{ce}}$) loss:


(8) \begin{equation*} \mathcal{L}={\mathcal{L}}_{\mathrm{cl}}+{\mathcal{L}}_{\mathrm{ce}}^{(1)}+{\mathcal{L}}_{\mathrm{ce}}^{(2)} \end{equation*}

### Implementation details and hyperparameters

To ensure the robustness and optimal configuration of the PhoSARte framework, we utilized five-fold cross-validation. This means that 80% of the training dataset is used for training, and the remaining 20% is used for validation in each fold. The optimal hyperparameters were determined through this rigorous process. The Adam optimizer [42] with a weight decay of $5\times10^{-5}$ and a learning rate of $1\times10^{-4}$, a batch size of 128, a number of epochs of 30, a dropout rate of 0.3, and a contrastive learning margin of 2 were also applied to enhance model performance and prevent overfitting. Detailed configurations for the SCL and PLM modules are available in Supplementary Tables S1 and S2.

### Performance evaluation metrics and statistical analysis

To evaluate the effectiveness and robustness of the proposed method, we employed a set of standard performance metrics commonly used for binary classification [36,43–46]. These metrics provide quantitative measures of model quality during training and testing. During training, the performance metrics computed via five-fold cross-validation were used to identify the best configuration and corresponding hyperparameters. Specifically, the metrics were calculated for each fold and then averaged across all five folds. This strategy was adopted instead of selecting a model based on a single best-performing fold, as it provides a more robust and generalizable assessment of model performance. In contrast, metrics computed on testing were used to assess model generalizability and facilitate fair comparisons with existing state-of-the-art methods on benchmark datasets. The performance was evaluated using seven metrics, namely accuracy (ACC), balanced ACC (BACC), specificity (Sp), sensitivity (Sn), Matthews correlation coefficient (MCC), the area under the receiver operating characteristic curve (AUC), and the area under the precision-recall curve (AUPRC). The formulas for these metrics are presented in Supplementary Note S3.

To assess whether the performance improvement achieved by PhoSARte over existing state-of-the-art methods was statistically significant, we employed a paired bootstrap test. Notably, this resampling technique compares two models’ performance on identical test datasets [47,48]. Specifically, we performed bootstrap resampling with replacement for 10 000 iterations to estimate the distribution of performance differences between PhoSARte and each competing method. A *P*-value of <.05 was considered statistically significant.

## Results and discussion

### Evaluation of protein language model embeddings in the PhoSARte framework on the A549 dataset

**Figure 2:**
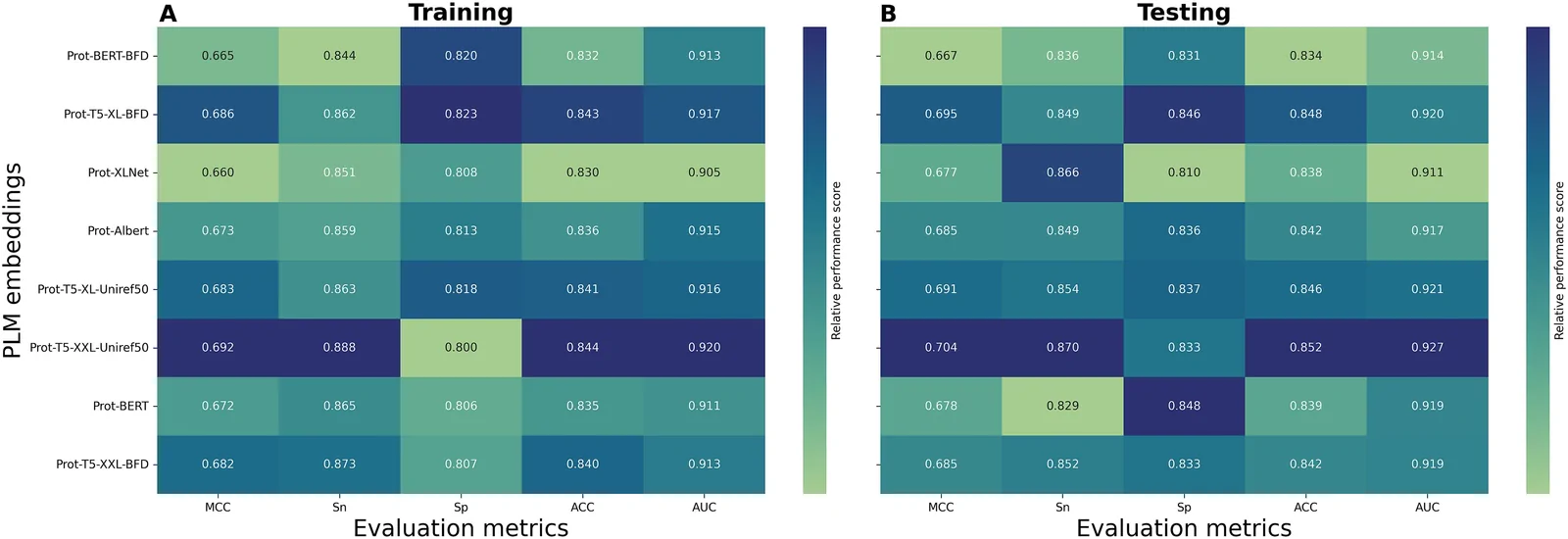
Performance comparison of the proposed method on the A549 training (A) and testing (B) datasets across various configurations. Heatmap plots showing each configuration’s (y-axis) performance across all metrics (x-axis). The colors are normalized per metric, with darker shades indicating higher scores. MCC: Matthews correlation coefficient; Sn: sensitivity; Sp: specificity; ACC: accuracy; AUC: area under the receiver operating characteristic curve.

To identify the most effective PLM embedding for detecting S/T phosphorylation sites, we systematically evaluated PhoSARte performance across eight different PLMs integrated with the SCL module (see **Materials and methods** section). On the training dataset (Fig. 2A), PhoSARte performance varied moderately depending on the PLM embeddings, with MCC values ranging from 0.660 to 0.692. Among all configurations, Prot-T5-XXL-Uniref50 achieved the best overall performance across all metrics, with MCC, ACC, Sn, Sp, and AUC values of 0.692, 0.844, 0.888, 0.800, and 0.920, respectively. This represents a significant improvement of 0.63%–3.19% in MCC, 0.16%–1.44% in ACC, and 0.30%–1.44% in AUC compared to other configurations. Although models using Prot-T5-XL-BFD, Prot-T5-XL-Uniref50, and Prot-T5-XXL-BFD also showed competitive performance, Prot-T5-XXL-Uniref50 consistently achieved the most balanced and improved performance, indicating its superior ability to encode phosphorylation-relevant sequence features. To assess transferability, we evaluated the performance of these models on an A549 testing dataset (Fig. 2B). Interestingly, the performance trend on this testing set was consistent with the training results, indicating that the models were trained effectively without overfitting. Remarkably, Prot-T5-XXL-Uniref50 achieved the best performance, with MCC, ACC, Sn, Sp, and AUC values of 0.704, 0.852, 0.870, 0.833, and 0.927, respectively. Based on these results, we selected the model with SCL and Prot-T5-XXL-Uniref50 as the top performer for the A549 dataset, which we designated PhoSARte_A549. The comparative analysis highlights that Prot-T5-XXL-Uniref50 is the most effective embedding for phosphorylation site prediction in SARS-CoV-2-infected A549 cells when combined with SCL. Its superior performance is likely due to its pretraining on a massive and diverse protein corpus, which enables it to generate a richer, more contextual understanding of protein sequences and leads to better generalization.

### Benchmarking protein language model variants in the PhoSARte framework using the Vero E6 dataset

**Figure 3:**
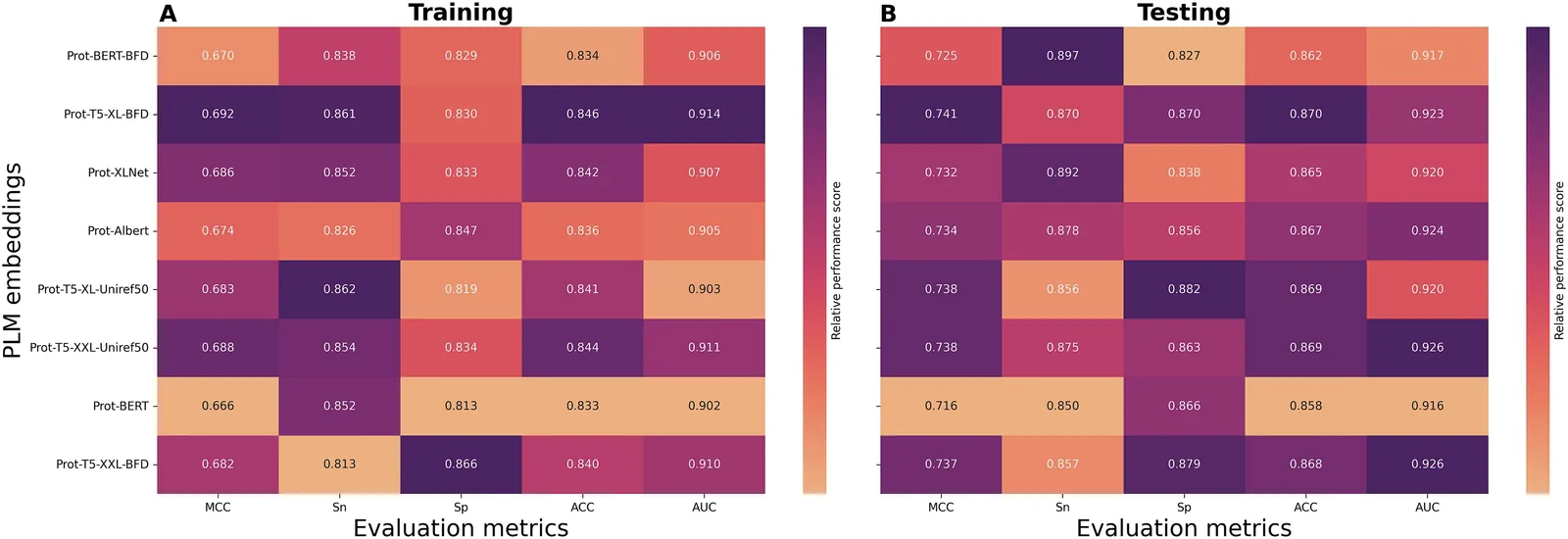
Performance comparison of the proposed method on the Vero E6 training (A) and testing (B) datasets across various configurations. Heatmap plots showing performance evaluation metrics (x-axis) of different PLM embeddings (y-axis), with darker shades indicating higher scores. MCC: Matthews correlation coefficient; Sn: sensitivity; Sp: specificity; ACC: accuracy; AUC: area under the receiver operating characteristic curve.

We used the same evaluation strategy on the Vero E6 training and testing datasets to benchmark the performance of PhoSARte with different PLMs, each integrated into the SCL module (Fig. 3). During training, Prot-T5-XXL-BFD and Prot-T5-XL-Uniref50 achieved a similar level of performance on global metrics (MCC and ACC). However, the model combining SCL with Prot-T5-XL-BFD achieved the highest training performance with MCC, Sn, Sp, ACC, and AUC of 0.692, 0.861, 0.830, 0.846, and 0.914, respectively. On the testing dataset, model performance varied depending on the PLM employed, with MCC ranging from 0.716 to 0.741, highlighting its complexity and inherent variability of phosphorylation sites in this cellular context.

Interestingly, the two models utilizing Prot-T5-XXL-BFD and Prot-T5-XL-Uniref50 showed similar performance on the training dataset, likely due to their shared architectural depth and training on the same protein corpora. However, their performance was less consistent on unseen data. In contrast, the model integrating SCL with Prot-T5-XL-BFD achieved the best performance on the testing dataset, with MCC, Sn, Sp, ACC, and AUC values of 0.741, 0.870, 0.870, 0.870, and 0.923, respectively. Notably, it outperformed other models by 0.27%–2.47% in MCC, 0.15%–1.24% in ACC, and 0.25%–0.72% in AUC. As a result, this model was chosen as the final model for the Vero E6 datasets and named PhoSARte_VeroE6.

### Cross-cell-type evaluation

To evaluate the reliability and generalizability of PhoSARte, we performed extensive *in silico* benchmarking using a cross-cell-type validation strategy. Specifically, models trained on one infected-cell dataset were tested on another biologically distinct infected-cell dataset after sequence redundancy reduction. For comprehensive comparison, we also included previously reported cross-cell-type results, such as DeepIPs, MeL-STPhos_2 (MeL-STPhos_A549), and MeL-STPhos_3 (MeL-STPhos_VeroE6), along with accessible web servers, trained models, or released weights, such as IPs-GRUAtt, PSPred-ALE, and PTransIPs (Table 1). It should be noted that DeepIPs, IPs-GRUAtt, PSPred-ALE, and PTransIPs were trained only on the A549 dataset. When models trained on the A549 dataset were evaluated on the Vero E6 dataset, PhoSARte_A549 achieved the highest performance in terms of ACC and MCC scores of 0.843 and 0.660, respectively. Specifically, PhoSARte_A549 improved ACC by 2.22%–7.28% and MCC by 4.35%–12.57% compared to other A549-specific models. Conversely, when the models trained on the Vero E6 dataset were evaluated on the A549 dataset, PhoSARte_VeroE6 outperformed MeL-STPhos_VeroE6, with improvements of 2.74% in ACC and 5.01% in MCC. These results firmly support the robustness and transferability of PhoSARte across biologically distinct cellular contexts. Both PhoSARte_A549 and PhoSARte_VeroE6 maintained similar performance levels across cell lines and outperformed existing methods in the cross-cell-type evaluation. Although future validation across additional cell lines and species can further expand its scope, this rigorous cross-evaluation demonstrates that our framework captures fundamental and universal patterns of S/T phosphorylation under SARS-CoV-2 infection conditions, rather than merely learning cell-specific features. Overall, these findings confirm that PhoSARte reliably captures phosphorylated sequence patterns that are not limited to a single cell type, successfully validating the tool for downstream biological applications and providing a rationale for future development of a unified model trained on integrated data from multiple cell lines and species.

**Table 1:** Performance comparison between cell line-specific PhoSARte models and state-of-the-art methods in cross-dataset evaluation.

**Dataset**	**Model**	**MCC**	**Sn**	**Sp**	**ACC**	**BACC**	**AUC**	**AUPR**
A549	MeL-STPhos_VeroE6	0.417	0.613	0.797	0.705	0.705	0.771	0.746
	PhoSARte_VeroE6	0.467	0.682	0.783	0.732	0.732	0.806	0.795
Vero E6	DeepIPs	0.534	0.799	0.750	0.782	0.775	0.847	0.907
	IPs-GRUAtt	0.536	0.746	0.816	0.770	0.781	0.854	0.907
	PSPred-ALE	0.557	0.815	0.756	0.795	0.785	0.850	0.902
	PTransIPs	0.616	0.828	0.805	0.820	0.817	0.888	0.934
	MeL-STPhos_A549	0.556	0.826	0.738	0.796	0.782	0.857	0.909
	PhoSARte_A549	0.660	0.855	0.819	0.843	0.837	0.887	0.925

MCC: Matthews correlation coefficient; Sn: sensitivity; Sp: specificity; ACC: accuracy; BACC: balanced ACC; AUC: area under the receiver operating characteristic curve; AUPRC: area under the precision-recall curve.

### Development of PhoSARte generic model

**Figure 4:**
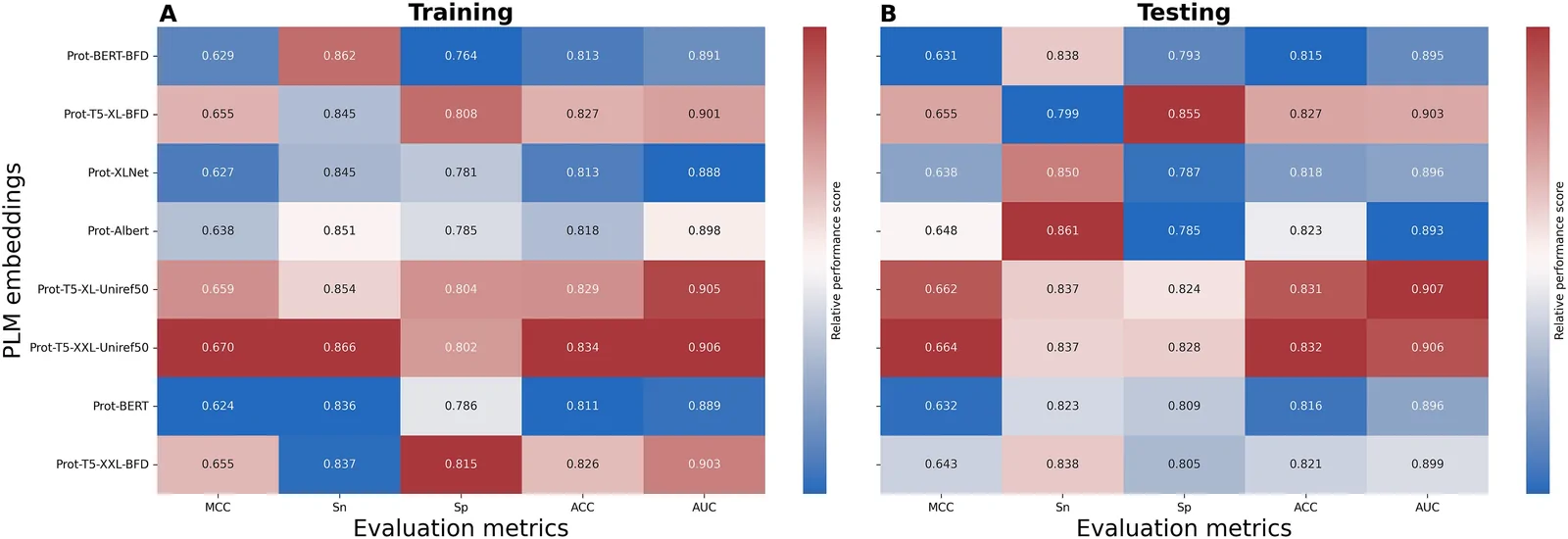
Performance comparison of the proposed method on the Combined training (A) and testing (B) datasets across various configurations. Heatmap plots showing the performance evaluation metrics (x-axis) of the proposed framework using different PLM embeddings (y-axis), with red indicating higher scores and blue indicating lower scores. MCC: Matthews correlation coefficient; Sn: sensitivity; Sp: specificity; ACC: accuracy; AUC: area under the receiver operating characteristic curve.

We constructed the Combined dataset by merging the A549 and Vero E6 cell data. We then evaluated all eight PLMs with SCL on this unified dataset (Fig. 4). Figure 4A shows that models incorporating Prot-T5 embedding variants demonstrated superior performance. Notably, the model using Prot-T5-XXL-Uniref50 achieved the best performance, with MCC, Sn, Sp, ACC, and AUC values of 0.670, 0.866, 0.802, 0.834, and 0.906, respectively. Consistent with the previous analysis, we evaluated these models on the Combined testing dataset (Fig. 4B). The model trained with SCL and Prot-T5-XXL-Uniref50 demonstrated exceptional performance, consistently achieving the highest score on the testing set. With an MCC of 0.664, an Sn of 0.837, an Sp of 0.828, an ACC of 0.832, and an AUC of 0.906, this model was selected as the final version for the combined dataset and designated PhoSARte_Generic for subsequent analysis.

### Performance comparison between PhoSARte and existing state-of-the-art methods

To rigorously evaluate the effectiveness and robustness of our proposed method, we benchmarked PhoSARte models against all available state-of-the-art methods on the same testing datasets (Table 2). Notably, all performance metrics of the compared methods were recalculated based on predictions generated from their respective web servers or standalone programs. Consequently, we excluded tools for which trained models or weights were unavailable, as well as those with inaccessible web servers. Detailed information about the web servers and standalone programs used for comparison is provided in Supplementary Table S3. Across all three datasets, PhoSARte consistently outperformed the existing methods, demonstrating superior predictive performance and robustness.

**Table 2:** Performance comparison between PhoSARte models and state-of-the-art methods on the testing datasets.

**Dataset**	**Method**	**MCC**	**Sn**	**Sp**	**ACC**	**AUC**	**AUPRC**	***P*-value**
Combined	MeL-STPhos_Generic	0.627	0.733	0.886	0.811	0.901	0.884	<.05
	PhoSARte_Generic	0.664	0.837	0.828	0.832	0.906	0.894	NA
A549	DeepIPs	0.613	0.802	0.811	0.806	0.894	0.888	<.001
	IPs-GRUAtt	0.692	0.838	0.855	0.846	0.919	0.917	<.05
	PSPred-ALE	0.665	0.83	0.834	0.832	0.910	0.915	<.05
	PTransIPs	0.670	0.839	0.831	0.835	0.916	0.921	<.05
	MeL-STPhos_A549	0.673	0.845	0.828	0.836	0.913	0.912	<.05
	PhoSARte_A549	0.704	0.870	0.833	0.852	0.927	0.930	NA
Vero E6	MeL-STPhos_VeroE6	0.708	0.817	0.889	0.853	0.925	0.903	<.05
	PhoSARte_VeroE6	0.741	0.870	0.870	0.870	0.923	0.903	NA

MCC: Matthews correlation coefficient; Sn: sensitivity; Sp: specificity; ACC: accuracy; AUC: area under the receiver operating characteristic curve; AUPRC: area under the precision-recall curve; NA: not applicable.

On the A549 dataset, PhoSARte_A549 achieved notable improvements of 1.20%–9.10% in MCC, 0.60%–4.60% in ACC, 0.80%–3.30% in AUC, 0.90%–4.20% in AUPRC, 2.50%–6.80% in Sn, and 0.20%–2.20% in Sp compared to all other methods. Among the compared methods, IPs-GRUAtt and PTransIPs achieved exceptional performance, with MCC values of 0.692 and 0.673, respectively. These results suggest that models employing DL-based feature extraction, whether through BiGRU or transformer-based DL approaches, can lead to better performance than traditional methods. On the Vero E6 dataset, PhoSARte_VeroE6 was compared with MeL-STPhos_VeroE6, the only existing method trained and evaluated on the same Vero E6 dataset. Notably, PhoSARte_VeroE6 outperformed MeL-STPhos_VeroE6 in most performance evaluation metrics, with an improvement of 3.30% in MCC, 1.70% in ACC, and 5.30% in Sn. Furthermore, on the Combined dataset, PhoSARte_Generic outperformed MeL-STPhos_Generic (MeL-STPhos_1) in nearly all performance metrics, with increases of 3.70% in MCC, 2.10% in ACC, 10.40% in Sn, 0.50% in AUC, and 1.00% in AUPRC. Although MeL-STPhos_Generic showed slightly better recognition of non-phosphorylation sites, PhoSARte_Generic demonstrated markedly improved detection of phosphorylation events, reinforcing its practical utility in SARS-CoV-2-related phosphoproteomics analysis. To further assess the reliability of these performance gains, we conducted paired bootstrap tests for the comparison, and the results are shown in Table 2. These results confirmed that the improvements achieved by PhoSARte models were statistically significant (*P*-values <.05, based on paired bootstrap tests) on the corresponding datasets. Collectively, these findings underscore PhoSARte’s superior accuracy, generalizability, and biological relevance, and further demonstrate that integrating SCL with PLM embeddings enables the extraction of meaningful sequence representations capable of distinguishing phosphorylation sites from non-phosphorylation sites across diverse cellular contexts.

### Case study on adenovirus type 2-infected human IMR-90 cells

To demonstrate that the utility of PhoSARte extends beyond SARS-CoV-2 infection, we evaluated its transferability to an entirely unseen infection dataset. Specifically, we conducted a cross-viral case study using human IMR-90 cells infected with adenovirus type 2 [49], a biologically distinct system from SARS-CoV-2 infection. After curation and redundancy reduction with a CD-HIT sequence identity threshold of 70% as well as a data filtering step, the final dataset comprised 2344 positive and 10 495 negative samples. As shown in Table 3, both generic and cell line-specific PhoSARte models were benchmarked against several state-of-the-art predictors.

**Table 3:** Performance comparison between PhoSARte models and state-of-the-art methods on the case study dataset.

**Method**	**MCC**	**Sn**	**Sp**	**ACC**	**BACC**	**AUC**	**AUPR**
MeL-STPhos_Generic	0.775	0.845	0.950	0.931	0.897	0.958	0.866
PhoSARte_Generic	0.801	0.898	0.945	0.937	0.922	0.967	0.891
DeepIPs	0.599	0.840	0.854	0.851	0.847	0.923	0.764
IPs-GRUAtt	0.608	0.798	0.879	0.864	0.838	0.916	0.755
PSPred-ALE	0.601	0.833	0.857	0.853	0.845	0.920	0.781
PTransIPs	0.682	0.859	0.897	0.890	0.878	0.942	0.850
MeL-STPhos_A549	0.690	0.867	0.898	0.892	0.882	0.944	0.853
PhoSARte_A549	0.668	0.882	0.878	0.878	0.880	0.940	0.816
MeL-STPhos_VeroE6	0.499	0.728	0.841	0.820	0.784	0.863	0.579
PhoSARte_VeroE6	0.604	0.846	0.852	0.851	0.849	0.918	0.753

MCC: Matthews correlation coefficient; Sn: sensitivity; Sp: specificity; ACC: accuracy; BACC: balanced ACC; AUC: area under the receiver operating characteristic curve; AUPRC: area under the precision-recall curve.

Table 3 shows that PhoSARte_Generic achieved the highest performance across nearly all evaluation metrics, with MCC, Sn, ACC, BACC, AUC, and AUPRC of 0.801, 0.898, 0.937, 0.922, 0.967, and 0.891, respectively. The only exception was Sp (0.945), which was slightly lower than that of the strong baseline MeL-STPhos_Generic (Sp = 0.950). These results indicate that PhoSARte_Generic provides a well-balanced and robust predictive performance. Compared with other generic and cell line-specific predictors, PhoSARte_Generic improved MCC by 2.68%–30.25%, ACC by 0.58%–11.64%, BACC by 2.44%–13.74%, AUC by 0.89%–10.39%, and AUPRC by 2.41%–31.19%, further confirming the effectiveness of integrating SCL and PLM representations for capturing phosphorylation-related sequence features. Collectively, these findings demonstrate that the generic model has strong generalizability and robustness across diverse biological contexts, including both SARS-CoV-2- and adenovirus type 2-infected cells.

In contrast, the performance of the A549-specific and Vero E6-specific models declined markedly compared with that of the generic models. Although PhoSARte_VeroE6 significantly outperformed our previous method, MeL-STPhos_VeroE6, PhoSARte_A549 ranked third in terms of MCC on the A549 dataset. Overall, the findings indicate that generic models provide the most reliable and transferable performance for phosphorylation site prediction in pathogen-perturbed cellular conditions, while cell line-specific models show variations in predictive ability influenced by both experimental context and dataset origin. Importantly, evaluation on this entirely unseen cross-viral dataset confirms the robustness and adaptability of PhoSARte across diverse viral species, providing rigorous computational evidence for its broader predictive capabilities. Beyond establishing this high predictive accuracy, PhoSARte also offers biological value by enabling the prioritization of high-confidence phosphorylation sites under novel viral stress conditions. As demonstrated in this analysis, researchers can use the PhoSARte web server to uncover previously uncharacterized virus-induced phosphorylation events under novel cellular stress conditions. Ultimately, identifying these specific host-kinase and viral-substrate interactions provides direct biological insight into viral pathogenesis and significantly accelerates the discovery of novel host-directed therapeutic targets, establishing PhoSARte as a powerful and highly adaptable framework for phosphorylation site prediction across various virus-infected cellular environments.

### Model interpretation and ablation analyses of PhoSARte

#### Visualization of learned features

**Figure 5:**
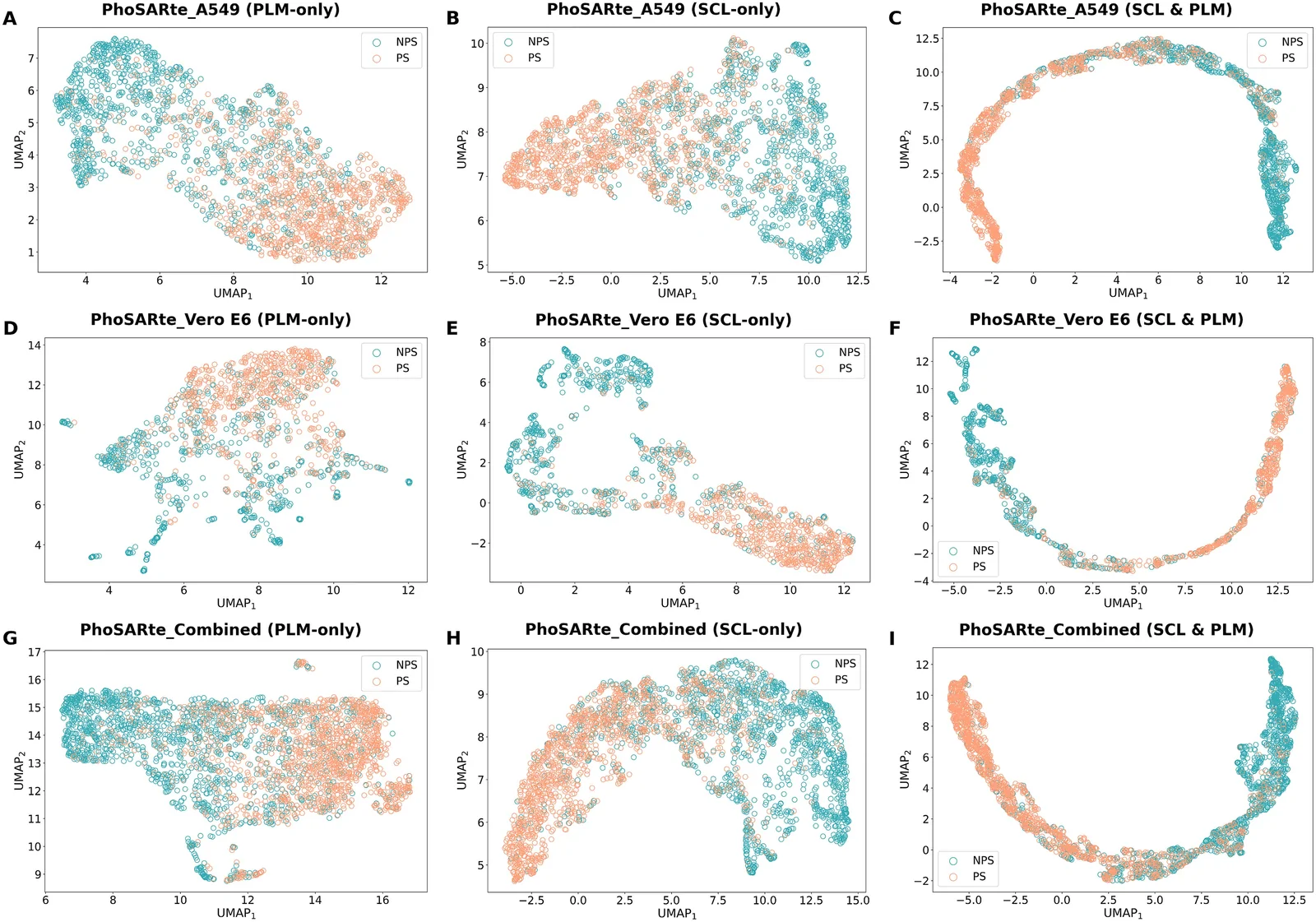
UMAP visualization of the features learned by different modules of the PhoSARte model: (A, D, and G) PLM-only module, (B, E, and H) SCL-only module, and (C, F, and I) PhoSARte. NPS: non-phosphorylated sites; PS: phosphorylated sites. UMAP: uniform manifold approximation and projection.

To gain deeper insight into the internal feature representations learned by different modules of the PhoSARte framework, we visualized the extracted features across three benchmark datasets (A549, Vero E6, and Combined). Notably, we used uniform manifold approximation and projection (UMAP) [50] to reduce the extracted high-dimensional feature space into two dimensions, facilitating qualitative analysis of feature clustering relative to phosphorylation sites. Figure 5 clearly shows that the feature representations extracted by the PLM module have significant overlap between phosphorylated and non-phosphorylated sites (Fig. 5A, D, and  G). This indicates that although PLMs capture rich contextual information from protein sequences, this information is not inherently structured to discriminate phosphorylation sites. In contrast, the features extracted by the SCL module show clearer separation between them, indicating their ability to maximize the distance between positive and negative samples, which is essential for the PhoSARte framework (Fig. 5B, E, and  H). Importantly, the combined feature representations of the SCL and PLM modules showed a significant discriminative separation from either module alone (Fig. 5C, F, and  I). This finding provides strong visual proof that the fusion of these two approaches is not merely additive but synergistic. The PLM module provides the rich sequence context, while the SCL module transforms this context into highly separable representations, leading directly to the superior predictive performance seen in our results. These observations were consistent across all three datasets, showing the robustness and generalizability of the PhoSARte framework for distinguishing between phosphorylated and non-phosphorylated sites. Overall, these results demonstrate that the PhoSARte framework learns meaningful, high-level features that are essential for robust and generalized phosphorylation site prediction, establishing its potential as a valuable tool for future research.

#### *In silico* mutagenesis analysis revealed key functional regions learned by PhoSARte

**Figure 6:**
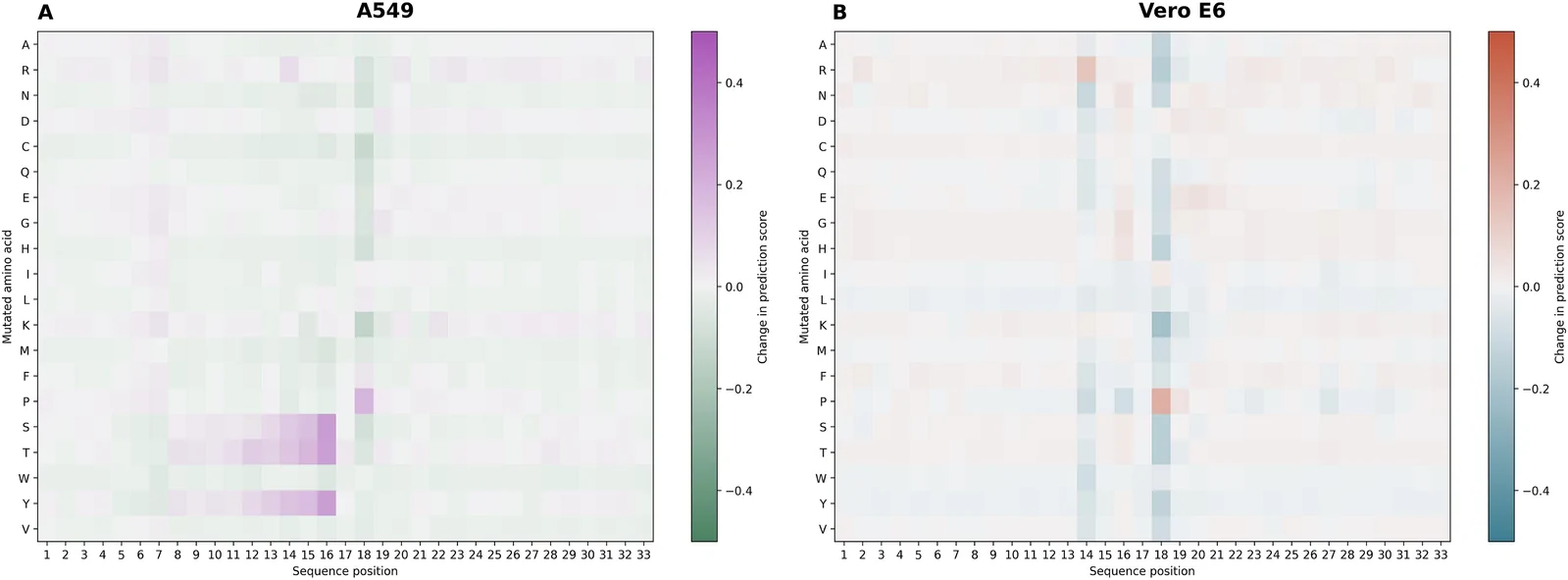
Heatmap visualization of the ISM analyses on the full sets of sequences from the A549 (A) and Vero E6 (B) testing datasets. The x-axis shows sequence positions (1–33), and the y-axis displays the mutated amino acids. Each cell represents the change in phosphorylation prediction score when the original residue at a given sequence position is substituted with one of the remaining 19 standard amino acids. Positive values indicate mutations that increase the predicted probability of phosphorylation, whereas negative values indicate disruptive substitutions. ISM: *in silico* mutagenesis.

To investigate the sequence determinants underlying PhoSARte’s predictions, we performed ISM analyses across all sequences in the testing datasets. For each sequence, every residue position was systematically substituted with the remaining 19 standard amino acids to generate all possible variants. PhoSARte models were used to compute the predictions, and the resulting changes in PhoSARte’s prediction scores relative to the corresponding wild-type sequences were recorded. These mutational effects were then averaged across all test sequences to generate residue-by-position heatmaps for the A549 and Vero E6 datasets (Fig. 6). To further examine class-specific trends, we also performed separate ISM analyses on the individual positive and negative samples; the corresponding results are provided in Supplementary Table S4 and Supplementary Figs S1 and S2.

On the A549 testing dataset, the ISM heatmap shows clear sensitivity at residues flanking the phosphorylation site, particularly at positions 14 (−3 relative to the phosphorylation site) and 18 (+1) (Fig. 6A). Notably, substitutions involving proline (P) at the downstream +1 position significantly affect the prediction score, consistent with the known proline-directed motif ([S/T]P) recognized by kinase families such as MAPK and CDK [7, 13, 14]. In parallel, substitutions at the upstream −3 position show increased sensitivity to basic residues such as lysine (K) and arginine (R), suggesting that PhoSARte also captures features associated with basophilic kinase motifs, such as RXX[S/T], which are commonly targeted by kinases such as PKA, PKC, and AKT [7, 13, 14]. These sequence preferences are in close agreement with experimentally characterized phosphorylation-associated motifs reported in curated resources, including PhosphoSitePlus^®^ [51] and Phospho.ELM [52]. Overall, these results suggest that PhoSARte learns biologically meaningful local sequence context surrounding phosphorylation sites rather than relying solely on the presence of the phospho-acceptor residue.

Consistent with the A549 dataset, the ISM heatmap on the Vero E6 dataset shows strong mutational sensitivity around the phosphorylation site (Fig. 6B), again with the most prominent effects centered at positions 14 (−3) and 18 (+1). Specifically, substitutions involving P at the downstream +1 position produce significant effects in the model output, in line with the well-known proline-directed phosphorylation motif. Additionally, substitutions involving R/K at the upstream −3 position are associated with strong sensitivity consistent with basophilic kinase motifs. These analyses indicate that PhoSARte relies primarily on the local sequence context immediately surrounding the phosphorylation site, rather than diffuse patterns distributed across the entire sequence length.

The class-specific analyses further clarified this behavior. In the A549-positive subset (Supplementary Fig. S1A), perturbation of position 18 (+1) consistently decreased the prediction score, highlighting the functional importance of this site in true positive sequences. In the corresponding A549-negative subset (Supplementary Fig. S1B), strong score increases were observed when substitutions were introduced at positions 14 to 16 (−3 to −1), particularly for residues occupying the immediate upstream region, while additional gains were also evident at position 18 (+1). In the Vero E6-positive subset (Supplementary Fig. S2A), the strongest negative effects were again concentrated at position 18 (+1), with weaker but detectable sensitivity at position 14 (−3). By contrast, the Vero E6-negative subset (Supplementary Fig. S2B) showed highly specific gain-of-score events, most notably for R/K at position 14 (−3) and P at position 18 (+1). These patterns are consistent with known phosphorylation-associated sequence preferences, including basophilic upstream context and proline-favored downstream context. Collectively, these findings indicate that PhoSARte captures biologically meaningful positional dependencies and recognizes phosphorylation sites through context-aware sequence features, rather than relying solely on the identity of the central S/T residue.

Notably, many of the sequence patterns highlighted by ISM analyses are consistent with known kinase motifs, including motifs associated with the MAPK, CDK, and PKA/PKC families, which regulate cellular processes such as stress responses, immune signaling, and viral replication. Previous phosphoproteomic studies have reported extensive remodeling of host phosphorylation networks during COVID-19, including activation of MAPK and PI3K/AKT-related signaling pathways [13]. In this context, the motif signatures identified by our ISM analysis may reflect phosphorylation-related regulatory features involved in host responses to viral infection. Because PhoSARte successfully captured these biologically relevant sequence determinants, it provides a highly interpretable framework for investigating phosphorylation-dependent pathways. Consequently, researchers can directly leverage these insights to identify potential candidate host-directed therapeutic targets. Taken together, these findings strongly support the biological validity and interpretability of PhoSARte, confirming that integrating PLM-derived contextual embeddings with SCL enables the model to learn true biologically relevant sequence–context relationships associated with phosphorylation.

### Ablation study on the architecture of PhoSARte

PhoSARte was developed by integrating the SCL and PLM modules, whose learned representations were subsequently fed into the classification head for final prediction. To quantify the contribution of each component, we conducted an ablation study by comparing the full PhoSARte architecture with two reduced variants: PLM-only and SCL-only.

Among the two ablated variants, SCL-only consistently outperformed PLM-only across all datasets (Supplementary Fig. S3). On the training dataset, MCC improvements of 0.81%–3.51%, ACC increases of 0.21%–1.71%, and AUC gains of 0.94%–1.90% were observed. Similar trends were observed on the testing dataset, where SCL-only improved MCC by 2.23%–4.95%, ACC by 1.16%–2.47%, and AUC by 1.50%–2.02% relative to PLM-only. These findings suggest that the SCL module provides stronger discriminative learning capacity when evaluated independently.

Importantly, the full PhoSARte model consistently outperformed both ablated variants across all datasets. On the A549 dataset, PhoSARte achieved the best performance in both training and testing sets. In the testing set, PhoSARte reached an MCC of 0.704 compared with 0.624 for PLM-only and 0.646 for SCL-only, while also achieving the highest values for Sn, Sp, ACC, and AUC. A similar trend was observed on the Vero E6 dataset, where PhoSARte achieved an MCC of 0.741 and an AUC of 0.923 in the testing set, outperforming both ablated variants. This advantage was further confirmed on the Combined dataset, where PhoSARte again achieved the highest testing performance, including an MCC (0.664) and an AUC (0.906), whereas PLM-only and SCL-only produced lower MCC values of 0.592 and 0.623, respectively.

Overall, these results demonstrate that both modules contribute complementary information to PhoSARte. The PLM module captures rich contextual representations from protein sequences, whereas the SCL module enhances discriminative feature learning through contrastive representation alignment. Their integration, therefore, results in consistently improved predictive performance and better generalization across datasets.

### Residue-specific performance evaluation of PhoSARte

To examine whether model performance is influenced by the type of phosphorylated residue, we evaluated PhoSARte separately on serine (S) and threonine (T) phosphorylation sites across the A549, Vero E6, and Combined testing datasets (Supplementary Table S5). Overall, the results showed comparable performance between the two residue types across all datasets. In A549, PhoSARte achieved MCC values of 0.698 for S and 0.762 for T. In Vero E6, the model produced MCC values of 0.744 for S and 0.708 for T. A similar trend was observed in the Combined dataset, where MCC values were 0.655 and 0.752, and AUC values were 0.903 and 0.933, for S and T, respectively. Overall, these results indicate that PhoSARte maintains stable predictive performance across both residue types, indicating that the model captures generalizable phosphorylation-related sequence patterns rather than exhibiting residue-specificbias.

### Web server deployment and user instructions

To ensure PhoSARte is accessible to both experimentalists and computational researchers, we have developed a user-friendly web server, available at https://balalab-skku.org/PhoSARte/. This server enables prediction of S/T phosphorylation sites from protein sequences using one of the three PhoSARte models (A549, Vero E6, and Generic). Among these, PhoSARte_Generic is designated as the default due to its superior generalizability and consistent performance across different cell types, as demonstrated in our benchmarking experiments. The server accepts input in two formats: users may either paste sequences directly into a text input field or upload a file in standard FASTA format. Example sequences are provided to help users format their inputs correctly. Once the prediction process is complete, users can download the results in CSV format, which includes both predicted labels and their associated probability scores for further analysis and visualization. If users prefer to be notified when the prediction is complete, they can enter their email addresses in the provided mailbox on the PhoSARte web server. Finally, users can retrieve their results using the job ID search. Furthermore, to support long-term accessibility, we have made the standalone version of PhoSARte, along with the trained models, publicly available through a GitHub repository (https://github.com/cbbl-skku-org/PhoSARte/). This lets other researchers run predictions locally on additional or updated datasets using their own computational resources.

### Limitations and future work

PhoSARte demonstrated superior performance in predicting phosphorylation sites across various datasets, including SARS-CoV-2- and adenovirus type 2-infected cells. However, as with any computational framework, several limitations remain and promising avenues for future improvement exist. First, Y phosphorylation sites were excluded from this study due to their low abundance in the available datasets and their overall rarity in the phosphoproteome. Expanding future datasets with a large number of well-annotated Y phosphorylation sites would enable broader coverage of phosphorylation types and improve the applicability of the model. Second, the current version of PhoSARte was specifically designed to model phosphorylation under virus-infected cellular conditions, rather than to serve as a general-purpose predictor across diverse physiological or disease states. Finally, the field still lacks a standardized benchmark dataset for fair and comprehensive evaluation of existing phosphorylation site predictors. The availability of such a resource would be invaluable for systematically assessing the true strengths and weaknesses of current methods and for guiding the development of the next generation of predictive tools.

Looking forward, PhoSARte provides a highly adaptable methodological blueprint for future bioinformatics development. Because the dual-stream integration of PLMs and SCL is not restricted to a specific viral species, the framework has the potential to be rapidly extended to emerging viral infections, sequence-based function predictions, and viral severity predictions. The successful application of PhoSARte to adenovirus type 2-infected IMR-90 cells supports its broader utility for prioritizing phosphorylation events under novel viral stress conditions. This adaptability may be particularly valuable during future outbreaks or “Disease X” scenarios, where computational prioritization of host-directed therapeutic targets could accelerate downstream experimental investigation before extensive virus-specific datasets become available. To build upon this foundation, we plan to expand the benchmark dataset by curating additional experimentally validated phosphorylation sites from SARS-CoV-2, with particular focus on Y phosphorylation, which remains unexplored. Additionally, we aim to develop an improved version of PhoSARte capable of detecting general phosphorylation sites from protein sequences beyond viral contexts. We will also conduct further comprehensive analyses to benchmark and evaluate all existing tools for identifying protein phosphorylation sites in SARS-CoV-2-infected cells. Furthermore, in future studies, we plan to incorporate multimodal features by combining sequence-based representations with structural information, such as embeddings derived from AlphaFold [53] or ESMFold [54], to further improve phosphorylation site prediction.

## Conclusion

Identifying phosphorylation sites is a crucial step toward understanding the molecular mechanisms of SARS-CoV-2 infection and advancing the development of novel therapeutic strategies. Although advances in DL and PLMs have been made, current research still faces challenges in achieving robust and generalized performance across different cell types, especially under unseen viral-infected conditions. In this study, we presented PhoSARte, a web server built upon a dual-stream computational framework for identifying S/T phosphorylation sites in SARS-CoV-2-infected cells. By bridging pretrained PLMs with an AttBiGRU within an SCL module, PhoSARte overcomes key limitations of traditional approaches that rely mainly on handcrafted features or DL-based feature extraction. Extensive benchmarking across SARS-CoV-2-infected A549 and Vero E6 cells demonstrated PhoSARte’s superior accuracy, transferability, and robustness over existing state-of-the-art methods. Importantly, the PhoSARte_Generic model trained on multi-cell data achieved strong generalization across distinct cellular and viral contexts, highlighting its utility as a broad-spectrum phosphorylation site predictor. Furthermore, a cross-viral case study on an entirely unseen adenovirus type 2-infected IMR-90 dataset demonstrated that PhoSARte is not limited to SARS-CoV-2, but rather serves as a highly adaptable methodological blueprint capable of generating actionable hypotheses during novel viral outbreaks.

To address the black-box limitation of many ML-based and DL-based predictors, PhoSARte incorporates interpretation analyses. UMAP visualizations confirmed that the SCL and PLM modules work synergistically to create a highly discriminative feature space, while ISM analysis revealed biologically relevant motifs and key phosphorylation-associated residues learned by the model. These findings indicate that PhoSARte functions not only as an accurate predictor, but also as an interpretable framework for extracting biologically relevant sequence determinants of virus-associated phosphorylation. PhoSARte is freely available at https://balalab-skku.org/PhoSARte/, providing an accessible platform for phosphorylation site prediction, host–virus interaction analysis, and antiviral target prioritization.

**Key Points**

- We developed PhoSARte, an interpretable deep learning framework that integrates Siamese network-based contrastive learning with pretrained protein language models for virus-associated phosphorylation site prediction.
- Cross-cell-type benchmarking using SARS-CoV-2-infected A549 and Vero E6 datasets showed that PhoSARte significantly outperforms state-of-the-art methods.
- A cross-viral case study on adenovirus type 2-infected human IMR-90 cells demonstrated the adaptability of PhoSARte beyond SARS-CoV-2.
- PhoSARte incorporates *in silico* mutagenesis analysis to identify biologically meaningful sequence positions and kinase-associated motif patterns.
- Freely accessible via a web server and as a standalone version, PhoSARte supports host-directed therapeutic development and antiviral target prioritization.

## Supplementary Material

PhoSARte_SI_R2_Camera_Ready_bbag504

## Data Availability

The web server for PhoSARte, along with all datasets used in this study, is available at https://balalab-skku.org/PhoSARte/. The standalone version of PhoSARte, along with the trained models, is available at https://github.com/cbbl-skku-org/PhoSARte/.
