# Supplementary material for: PhoSARte: identification of SARS-CoV-2 phosphorylation sites using contrastive learning and protein language models
Source: Brief Bioinform. 2026 Sep 15;27(5):bbag504. doi: 10.1093/bib/bbag504 (PMC13577671; doi:10.1093/bib/bbag504)
Supplement: Supplementary file 1 [file phosarte_si_r2_camera_ready_bbag504.docx]

**Supplementary data**

**PhoSARte: Identification of SARS-CoV-2 phosphorylation sites using contrastive learning and protein language models**

Nhat Truong Pham^1,†^, Duong Thanh Tran^1,†^, Qiaosen Su^1^, Yeona Jung^1^, Nattanong Bupi^1^, Dahyun Kang^1^, Sukchan Lee^1,*^, and Balachandran Manavalan^1,*^

^1^Department of Integrative Biotechnology, College of Biotechnology and Bioengineering, Sungkyunkwan University, Suwon 16419, Gyeonggi-do, Republic of Korea

^†^ Nhat Truong Pham and Duong Thanh Tran contributed equally to this work.

^*^Corresponding authors

Sukchan Lee, Department of Integrative Biotechnology, College of Biotechnology and Bioengineering, Sungkyunkwan University, Suwon 16419, Gyeonggi-do, Republic of Korea. Tel: +82-31-290-7866; Fax: +82-31-290-7870; E-mail: cell4u@skku.edu

Balachandran Manavalan, Department of Integrative Biotechnology, College of Biotechnology and Bioengineering, Sungkyunkwan University, Suwon 16419, Gyeonggi-do, Republic of Korea. Tel: +82-31-299-4858; Fax: +82-31-290-7870; E-mail: bala2022@skku.edu

**Supplemental notes**

**Note S1. Data preparation for the contrastive learning architecture**

Unlike self-supervised approaches such as SimCLR [1] that rely on data augmentations to generate positive pairs, our method leverages supervised contrastive learning by explicitly using class labels to define positive (same-class) and negative (different-class) pairs. During training, mini-batches of batch size ($bs$) were randomly sampled from the training dataset using standard PyTorch shuffling ($shuffle=True$). Within each batch, we systematically paired the first half ($\frac{bs}{2}$) with the corresponding samples from the second half, creating exactly four sequence pairs per batch.

Pair labels were deterministically computed using an XOR operation on the ground-truth class labels: $pair\_label = label1 \wedge label2$, where $label1, label2 \in\{0, 1\}$. This yields $pair\_label = 0$ for positive pairs (same class) and $pair\_label = 1$ for negative pairs (different classes). For a balanced binary dataset, this sampling strategy produces approximately 50% positive and 50% negative pairs per batch on average, though the exact ratio varies stochastically across batches due to random sampling.

**Note S2. Attention-based BiGRU architecture**

The GRU’s architecture only consists of two gates: the update gate ($z_{t}$) and the reset gate ($r_{t}$), which are trained to selectively manage information flow and retain past information. At each time step $t$ (i.e, t-th residue in a sequence), the GRU’s output is computed as follows:

$$\begin{aligned} z_{t}=\sigma\left( W_{z}\cdot\left[ h_{t-1},s_{t} \right] \right)\#(1)\# \end{aligned}$$

$$\begin{aligned} r_{t}=\sigma\left( W_{r}\cdot\left[ h_{t-1},s_{t} \right] \right)\#(2)\# \end{aligned}$$

$$\begin{aligned} {\overset{̃}{h}}_{t}=\tanh\left( W\cdot\left[ r_{t}\circ h_{t-1},s_{t} \right] \right)\#(3)\# \end{aligned}$$

$$\begin{aligned} h_{t}=\left( 1-z_{t} \right)\circ h_{t-1}+z_{t}\circ{\overset{̃}{h}}_{t}\#(4)\# \end{aligned}$$

Here, $W_{z},W_{r}$ and$W$ represent weightparameters; $s_{t}$is the input; $h_{t}$ is the output; ${\overset{̃}{h}}_{t}$ is the candidate hidden state; σ and $\tanh$ are the sigmoid and Hyperbolic Tangent (Tanh) activation functions, respectively; the symbol ‘$\circ$’ is the Hadamard product operator. For $t=0$, the output vector is $h_{0}=0$. The BiGRU network is effective for extracting high-latent features because it processes sequences in both forward and backward directions. The final output at each time step $t$ ($t>0$) is a concatenation of the forward ($\vec{h}_{t}$) and backward (${\overset{⃖}{h}}_{t}$) hidden states, represented as $h_{t}=[\vec{h}_{t},{\overset{⃖}{h}}_{t}]$.

The mathematical equations for this attention mechanism are defined as follows:

(i) The weight score of the amino acid *t*-th in the protein sequence is calculated as:

$$\begin{aligned} \varepsilon_{t}=\mathbb{W}^{\top}\cdot h_{t}\#(5)\# \end{aligned}$$

(ii) Then, the normalized importance weight is calculated by a SoftMax function as:

$$\begin{aligned} \alpha_{t}=\frac{e^{\varepsilon_{t}}}{\sum_{j=1}^{L}e^{\varepsilon_{j}}}\#(6)\# \end{aligned}$$

(iii) Finally, the output is calculated by performing a weighted sum as:

$$\begin{aligned} c_{t}={\sum_{t=1}^{L}\alpha}_{t}h_{t}\#(7)\# \end{aligned}$$

**Note S3. Performance evaluation metrics**

The formulas for accuracy (ACC), balanced ACC (BACC), specificity (Sp), sensitivity (Sn), and Matthews correlation coefficient (MCC) are as follows:

$$\begin{aligned} ACC=\frac{TP+TN}{TP+TN+FP+FN}\#(8)\# \end{aligned}$$

$$\begin{aligned} Sp=\frac{TN}{TN+FP}\#\left( 9 \right) \end{aligned}$$

$$\begin{aligned} Sn=\frac{TP}{TP+FN}\#\left( 10 \right) \end{aligned}$$

$$\begin{aligned} BACC=\frac{Sn+Sp}{2}\#\left( 11 \right) \end{aligned}$$

$$\begin{aligned} MCC=\frac{TP\times TN-FP\times FN}{\sqrt{\left( TP+FP \right)\times\left( TP+FN \right)\times\left( TN+FP \right)\times\left( TN+FN \right)}} \#\left( 12 \right) \end{aligned}$$

Here, the number of correctly predicted positive and negative samples is called TP (true positives) and TN (true negatives), respectively. The number of positive samples incorrectly predicted as negative is called FN (false negatives), while the number of negative samples incorrectly predicted as positive is called FP (false positives).

**Supplemental tables**

**Table S1.** The configuration for the SCL module.

| **Hyper-parameter** | **Value** |
| --- | --- |
| Learning rate | 0.0001 |
| Weight decay | 5e-5 |
| Epochs | 30 |
| Batch size | 128 |
| Dropout rate | 0.3 |
| Vocabulary size | 22 |
| Embedding dimension of the embedding layer | 512 |
| Hidden dimension in GRU | 128 |
| Number of recurrent layers in GRU | 2 |
| Number of heads of attention in transformer encoder layer | [8, 16, 32] |
| Number of layers in transformer encoder | [1, 2, 4] |

**Table S2.** The configuration for the PLM module.

| **Hyper-parameter** | **Value** |
| --- | --- |
| Dimension of the PLM-based embeddings | {1024, 4096} |
| Hidden dimension in GRU | 128 |
| Number of recurrent layers in GRU | 1 |

**Table S3.** Detailed information about the web servers and standalone programs of the state-of-the-art methods.

| **Method** | **Link** |
| --- | --- |
| MeL-STPhos_Generic | https://balalab-skku.org/MeL-STPhos/ |
| MeL-STPhos_A549 | https://balalab-skku.org/MeL-STPhos/ |
| MeL-STPhos_VeroE6 | https://balalab-skku.org/MeL-STPhos/ |
| DeepIPs | http://lin-group.cn/server/DeepIPs/ |
| IPs-GRUAtt | http://cbcb.cdutcm.edu.cn/phosphory/ |
| PSPred-ALE | https://github.com/jiaoshihu/PSPred-ALE |
| PTransIPs | https://github.com/StatXzy7/PTransIPs |

**Table S4.** Samples from the A549 and Vero E6 testing datasets for ISM analysis.

| **Sample** | **Sequence** |
| --- | --- |
| A549 positive | ‘KTESASVQGRNTDVAQSPEAPKQEAPAKKKSGS’ |
| A549 negative | ‘DSNLKPEEVVHKEKRRTKSLLEEKLVLKSKSKT’ |
| Vero E6 positive | ‘RKKGQHGSEHSKSRSSSPGKPQAVSSLNSSHSR’ |
| Vero E6 negative | ‘MFATSGAVAAGKPYSCSECGKSFCYSSVLLRHE’ |

**Table S5.** Performance of PhoSARte based on phosphorylated sites on the testing datasets.

| **Dataset** | **Method** | **MCC** | **Sn** | **Sp** | **ACC** | **AUC** | **AUPRC** |
| --- | --- | --- | --- | --- | --- | --- | --- |
| A549 | PhoSARte_A549_S | 0.698 | 0.870 | 0.828 | 0.849 | 0.926 | 0.929 |
|  | PhoSARte_A549_T | 0.762 | 0.876 | 0.886 | 0.881 | 0.929 | 0.934 |
| Vero E6 | PhoSARte_VeroE6_S | 0.744 | 0.873 | 0.871 | 0.872 | 0.922 | 0.899 |
|  | PhoSARte_VeroE6_T | 0.708 | 0.846 | 0.865 | 0.855 | 0.933 | 0.949 |
| Combined | PhoSARte_Generic_S | 0.655 | 0.833 | 0.822 | 0.827 | 0.903 | 0.890 |
|  | PhoSARte_Generic_T | 0.752 | 0.869 | 0.883 | 0.876 | 0.933 | 0.923 |

MCC: Matthews correlation coefficient; Sn: sensitivity; Sp: specificity; ACC: accuracy, AUC: area under the receiver operating characteristic (ROC) curve; AUPRC: Area under the precision-recall curve.

**Supplemental figures**

**Figure S1.** Heatmap visualization of the ISM analysis on individual sequences of the A549 testing dataset. **(A)** Heatmap visualization of the positive sequence (‘KTESASVQGRNTDVAQSPEAPKQEAPAKKKSGS’). **(B)** Heatmap visualization of the negative sequence (‘DSNLKPEEVVHKEKRRTKSLLEEKLVLKSKSKT’).

**Figure S2.** Heatmap visualization of the ISM analysis on individual sequences of the Vero E6 testing dataset. **(A)** Heatmap visualization of the positive sequence (‘RKKGQHGSEHSKSRSSSPGKPQAVSSLNSSHSR’). **(B)** Heatmap visualization of the negative sequence (‘MFATSGAVAAGKPYSCSECGKSFCYSSVLLRHE’).

**Figure S3.** Performance comparison between PhoSARte models and individual modules (PLM-only and SCL-only) on the training and testing datasets. (**A-B**) Performance comparison on the A549 training and testing sets. (**C-D**) Performance comparison on the Vero E6 training and testing sets. (**E-F**) Performance comparison on the Combined training and testing sets. MCC: Matthews correlation coefficient; Sn: sensitivity; Sp: specificity; ACC: accuracy, AUC: area under the receiver operating characteristic (ROC) curve.

**References**

1.	Chen T, Kornblith S, Norouzi M et al. A Simple Framework for Contrastive Learning of Visual Representations, International Conference on Machine Learning, Vol 119 2020;119.
